# Selected Deposition Techniques and the Effect of Doping on the Properties of Thin ZnO Films: A Literature Review

**DOI:** 10.3390/ma19091686

**Published:** 2026-04-22

**Authors:** Jakub Polis, Krzysztof Lukaszkowicz, Marek Szindler, Gabriela Wielgus, Julia Kolasa

**Affiliations:** 1 Department of Engineering Materials and Biomaterials, Faculty of Mechanical Engineering, Silesian University of Technology, Konarskiego 18a, 44-100 Gliwice, Poland; jakub.polis@polsl.pl; 2 Scientific and Didactic Laboratory of Nanotechnology and Material Technologies, Faculty of Mechanical Engineering, Silesian University of Technology, Towarowa 7, 44-100 Gliwice, Poland; marek.szindler@polsl.pl; 3 Department of Biomaterials and Medical Devices Engineering, Faculty of Biomedical Engineering, Silesian University of Technology, Roosevelta 40, 41-800 Zabrze, Poland; gabriela.wielgus@polsl.pl (G.W.); julia.kolasa@polsl.pl (J.K.)

**Keywords:** zinc oxide, thin films, synthesis, doped ZnO, TCO

## Abstract

Zinc oxide (ZnO) is currently one of the most significant wide-bandgap semiconductor materials, attracting extensive research across diverse fields including materials science, chemistry, physics, medicine, electronics, and power engineering. Its exceptional properties, such as high optical transparency, high electron mobility, chemical stability, and compatibility with low-cost fabrication techniques, have established ZnO as a versatile material with immense application potential. A critical application for ZnO is its role as a transparent conducting oxide (TCO) in modern optoelectronic and photovoltaic devices, as well as in sensors, transparent electronics, and spintronics. To meet the requirements of these advanced applications, precise control over the structural, optical, and electrical properties of ZnO thin films is essential. This is effectively achieved through the selection of specific synthesis methods and intentional modification techniques, such as doping. This review provides a comprehensive overview of the synthesis and modification of ZnO thin films, with a particular focus on how various dopants influence their fundamental characteristics. The work discusses a range of deposition techniques, including physical vapor deposition (PVD), chemical vapor deposition (CVD), atomic layer deposition (ALD), sol–gel methods, spray pyrolysis, and other solution-based approaches. The novelty of this review lies in its comparative analysis of different doping strategies combined with various thin-film deposition techniques, highlighting how specific synthesis routes influence dopant incorporation and ultimately determine functional properties. Furthermore, recent advances in tailoring ZnO thin films are summarized, alongside the identification of key challenges and future research directions. Ultimately, this work aims to provide researchers with a systematic perspective on the synthesis–structure–property relationships in doped ZnO thin films to support the development of optimized materials for next-generation electronic and optoelectronic devices. This review, thus, serves as a comprehensive reference for researchers and engineers seeking to optimize the functionality of ZnO-based thin films for emerging technological applications.

## 1. Introduction

Over a century ago, the first reports emerged suggesting that zinc oxide (ZnO) exhibits intriguing photochemical properties with potential applications in the field of energy. The term ‘photocatalysis’ was first introduced in 1911 by German chemist Alexander Eibner, who observed the bleaching of Prussian blue pigment under light exposure in the presence of ZnO [1,2]. In subsequent decades, research focusing on the photocatalytic oxidation of organic compounds (e.g., the conversion of phenol to catechol) and the utilization of metal oxides such as ZnO and TiO_2_ for the degradation of pollutants under UV irradiation contributed to the development of the concept of photomineralization and the advancement of photochemical applications for these materials [2,3,4].

**Figure 1:**
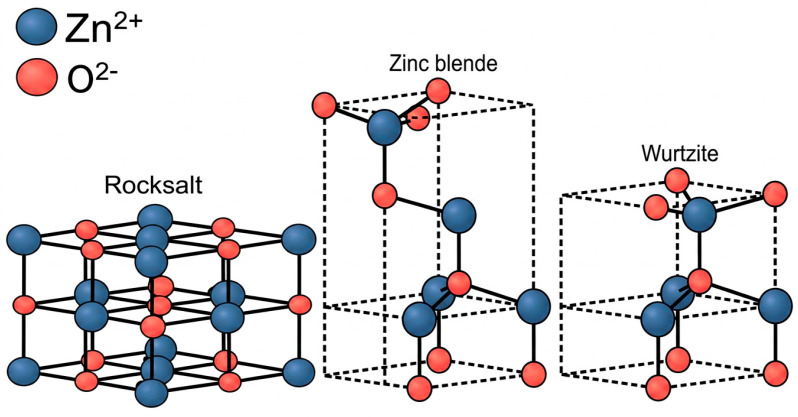
Crystallographic varieties of zinc oxide (based on [9]).

In nature, zinc oxide occurs as a rare mineral known as zincite, which crystallizes in the hexagonal wurtzite structure. This mineral was discovered in 1810 in the United States (Franklin, New Jersey). Zincite deposits are also found in Tuscany (Italy), Tsumeb (Namibia), and even in Poland, specifically in the Olkusz region. Naturally, it is white in color; however, it is frequently found to be contaminated with manganese, which imparts a red hue [5,6,7]. Zinc oxide can exist in three distinct crystalline structures. In addition to the aforementioned wurtzite, which is the thermodynamically stable phase at room temperature, ZnO also forms zinc blende (a metastable phase) and the rarely observed cubic rock salt structure, the stabilization of which requires the application of extreme pressure [5,6,7,8,9]. The crystallographic modifications of zinc oxide are illustrated in Figure 1.

Currently, zinc oxide is considered a multifunctional material due to its unique chemical and physical properties (Table 1). In 2025, the global zinc oxide market volume reached 2.36 million tons, and it is projected to reach 2.44 million tons in 2026 and 2.92 million tons by 2031 [10]. The primary application sector for zinc oxide (38.79%) is the rubber industry, where it serves as a vulcanization activator [10,11]. It is also employed in the modification of concrete, the most widely used construction material globally. Zinc oxide enhances concrete durability by improving its mechanical properties; moreover, its antibacterial, antiviral, and fungistatic activities can contribute to mitigating health risks for building occupants [12,13,14]. Along with titanium dioxide, zinc oxide is the only inorganic UV filter currently approved for use in sunscreens [15]. ZnO is also used in the food industry as a zinc supplement and nutrient fortifier, as well as an agricultural fertilizer [16]. From a public health standpoint, zinc oxide is an exceptionally versatile material used for applications including bioimaging [17], drug and gene delivery [18,19], inflammation reduction [20], and combating bacterial, viral, and fungal infections [21,22,23]. Additionally, it aids in the treatment of wounds [24], cancer [25], and potentially diabetes [26].

Zinc oxide is one of the most significant semiconductors. It finds applications in solar cells [27], gas sensors [28], catalysts [29], transistors [30], photodetectors [31], and memristors [32], as well as serving as a transparent conducting oxide (TCO) [33].

**Table 1:** Different properties of Zinc Oxide.

Properties	Value	References
Molecular weight	81.406 g/mol	[34]
Density	5.61 g/cm^3^	[35]
Tensile Modulus	250 GPa	[36]
Melting Point	1975 °C	[37]
Boiling point	2360 °C	[38]
Band Gap (room temperature)	3.37 eV	[39]
Exciton Binding Energy	60 meV	[40]
Electron Mobility	180 cm^2^/Vs	[41]
Refractive index	1.614	[38]
Thermal conductivity	50 W/(m × K)	[42]

**Figure 2:**
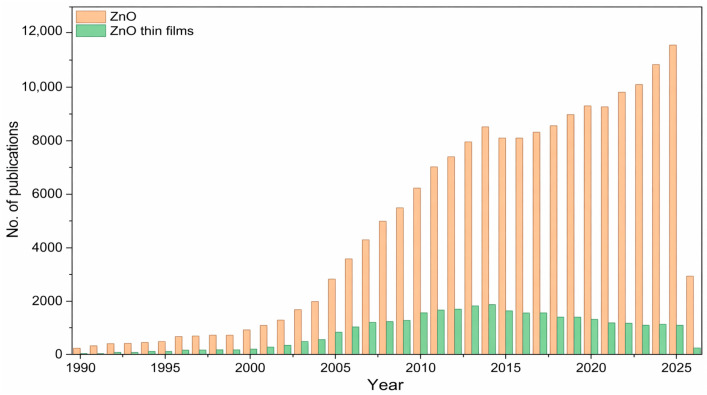
Evolution of the number of scientific papers related to the search for “ZnO” and “ZnO thin films” phrases published between 1990–2026. Inset: illustration of the last decade. Source: Scopus (accessed on 17 March 2026).

Transparent conducting oxides (TCOs) are a class of materials characterized by properties that are, to some extent, opposing: they simultaneously exhibit transparency in the visible light spectrum and electrical conductivity. Typically, transparent materials are classified as insulators, whereas high electrical conductivity is characteristic of metals and their alloys [43,44]. They constitute fundamental components in optoelectronic applications, ranging from solar cells [45], photocatalysts [46], and photodiodes [47] to semiconductor lasers [48], thin-film transistors [49], and gas sensors [50]. The primary group of materials utilized for TCO design consists of oxides with a wide bandgap E_g_ exceeding 3 eV, representing materials with high transparency for visible wavelengths. Zinc, indium, and tin oxides align perfectly with these requirements and demonstrate superior performance. In addition to the data provided in Table 1, ZnO is characterized by high transparency (>85%) and low resistivity, which can be reduced to values on the order of 10^−4^ Ω·cm. Furthermore, the natural abundance of zinc oxide and its relatively low cost are noteworthy [10,51,52,53,54]. To illustrate the increasing research interest in ZnO, Figure 2 presents a graph showing the annual number of publications on ZnO and ZnO thin films between 1990 and 2026, retrieved from the Scopus database.

The aim of this work is to discuss selected synthesis methods for zinc oxide thin films, their modification techniques, and to conduct a systematic analysis of the potential for enhancing the optical and electrical properties of these films.

## 2. Zinc Oxide Thin Film Deposition Methods

The optoelectrical properties of zinc oxide are strongly dependent on the fabrication method. The following chapter provides an overview of the most commonly utilised techniques for the preparation of zinc oxide thin films.

**Figure 3:**
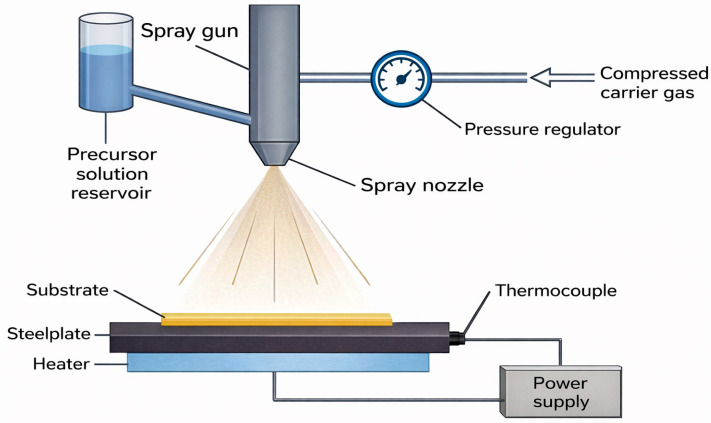
The process of producing a thin layer using spray pyrolysis (based on [57,63]).

One of the possible methods for fabricating ZnO thin films is spray pyrolysis (SP). This method involves the chemical synthesis of materials under atmospheric pressure, where a precursor solution of chemical compounds in a suitable solvent is atomized into small droplets through a nozzle onto a heated substrate (200–500 °C). The solvent evaporates, leaving behind a thin film of the desired material or a powder. Synthesis parameters are typically optimized for the required optical, structural, electrical, and mechanical properties of the materials. The thickness (typically 150–700 nm for ZnO thin films) and properties of the coating can be controlled by adjusting the precursor solution concentration, the flow rates of the solution and carrier gas, the spray rate, and the substrate temperature. This method is characterized by simplicity, reproducibility, and low cost. It does not require high-quality substrates and can operate within a moderate temperature range without the need for a vacuum. Compared to other synthesis methods, the spray pyrolysis method is efficient and relatively inexpensive. Additionally, this method enables the deposition of coatings on various types of substrates, including glass, metals, and polymers. ZnO coatings produced by this method exhibit high transparency (>95%), hardness, and excellent stability in neutral and alkaline aqueous environments. Furthermore, this method allows for the production of coatings with mobility reaching 100 cm^2^V^−1^s^−1^, electrical resistance of ≈40 Ω/cm^2^, and an optical bandgap within the range of 3.1–3.7 eV. However, the method has a significant limitation: the difficulty of obtaining a high-density, pore-free material [55,56,57,58,59,60,61,62,63,64]. A schematic of the method is presented in Figure 3.

Another method enabling the fabrication of ZnO thin films is chemical bath deposition (CBD). This process involves the deposition of inorganic thin films (oxides, sulfides, halides, etc.) at relatively moderate temperatures (<100 °C) by immersing the substrate in a precursor solution of the substance to be deposited. It is based on a controlled chemical reaction (usually hydrolysis), typically conducted in an aqueous solution, which results in the coating of the substrate (heterogeneous nucleation at the solution–substrate interface) while simultaneously leading to the formation of a colloidal suspension (homogeneous nucleation occurring in the solution). The aqueous precursor solution contains a soluble metal salt, a pH-regulating agent, and a complexing agent used to control the hydrolysis rate. During the deposition process, uniform stirring of the solution is necessary, for which a magnetic stirrer is employed. Usually, to regulate the reaction, precursor concentrations, pH, annealing conditions, and temperature are varied. As the reaction progresses, the thin film expands on the substrate layer by layer. Deposition conditions can be modified to monitor and regulate the growth process; for ZnO thin films, layers typically range from 100 to 500 nm in thickness [64,65,66,67,68].

**Figure 4:**
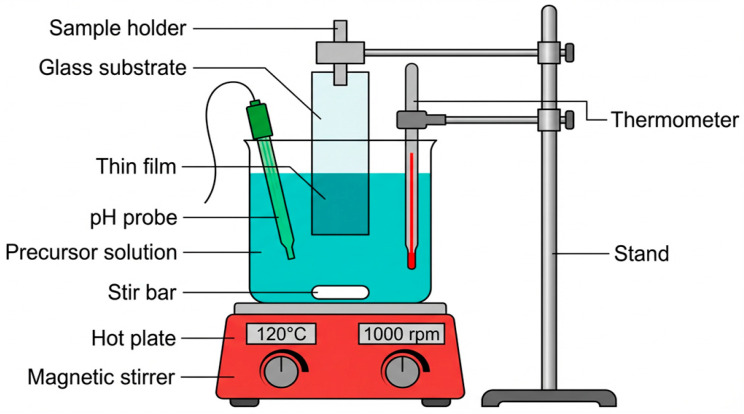
Schematic representation of the CBD process (based on [59,65,67]).

CBD is gaining importance due to its low cost, simplicity, ease of process control, wide choice of substrates, and the ability to produce uniform, compact coatings over large surface areas with good adhesion to the substrate. Furthermore, it is possible to deposit coatings on several elements in a single cycle. Generally, the CBD process depends on chemical reactions occurring at the solution–substrate interface. The reaction rate affects the structure and electrical properties of the thin film. However, the underlying mechanisms of the process and their methodical implementation require further research and remain a subject of scientific debate. The method also carries challenges, one of which is the appropriate selection of a substrate that does not react with the precursor solution. In the case of producing multilayer coatings, undesirable interactions may occur between previously deposited layers and the deposition solution. The CBD method is sensitive to minor fluctuations in reactant concentrations, pH, and temperature, which translates into variability in crystallisation, morphology, optical properties, and stoichiometry of the deposited layers, making it difficult to achieve reproducible results. Attention should also be paid to the potential environmental contamination risk posed by the used solution, which often contains toxic components. To mitigate these risks, it is recommended to employ filtration and chemical reactions of the precipitate with appropriate reagents, enabling the recovery of the starting material for reuse in the subsequent deposition stage [68,69,70]. A schematic of the method is presented in Figure 4.

In the case of the CBD process for depositing zinc oxide thin films, glass substrates are most commonly used and must be properly cleaned before the process begins. In publication [71], the authors obtained a thin zinc oxide film using a solution of zinc nitrate in distilled water and ammonia, resulting in nanocrystalline ZnO films with a wurtzite crystal structure. This coating showed low absorption in the visible range and higher absorption in the UV range. The hydrophobic nature of the resulting film was also confirmed. The authors of publication [72] synthesised ZnO thin films using zinc acetate dihydrate, triethanolamine, and ammonium hydroxide. The samples obtained were annealed at 400 °C and 600 °C, leaving a representative as-deposited (raw) sample unannealed. The results showed a slight difference in the bandgap values, which ranged from 3.20 to 3.23 eV. Annealing at 400 °C caused the agglomerates to become denser and adhere more strongly to the substrate, and a reduction in stress was achieved. Treatment at 600 °C led to a higher-quality, well-defined crystalline wurtzite ZnO structure. The results indicate that despite the stability of the energy gap, thermal treatment significantly improves structural ordering and the functional properties of the films. The authors of work [73] investigated the effect of pH on the fabrication of ZnO thin films via the CBD method. They demonstrated that the solution pH does not affect the crystallinity of the zinc oxide nanostructures.

**Figure 5:**
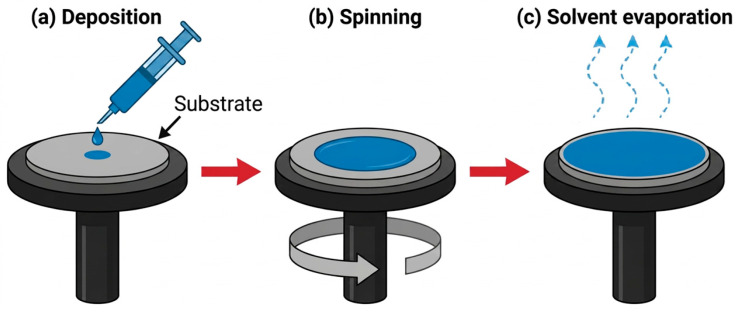
Schematic representation of the principle of the spin coating process for thin films (based on [74,75]).

Spin coating is a popular technique that has been successfully used to produce thin ZnO layers. This technique belongs to the broadly understood sol–gel method and is a solution-based deposition process. While it enables the production of uniform coatings of a specified thickness, material losses often exceed 90%, leading to a significant increase in material costs as the coated surface area increases. Nevertheless, the low requirement for the precursor solution and rapid solvent evaporation make it a widely used method for surface modification [74,75,76,77]. In the first step, the solution is applied to the centre of a flat substrate, which may rotate both before and after the solution application. The substrate is rotated at a specific spin speed. The rotation generates a centrifugal force that gradually spreads the solution toward the edges of the substrate, while simultaneous solvent evaporation occurs. High-volatility components are removed from the substrate through evaporation, while the low-volatility components of the solution remain on the substrate surface. Consequently, a uniform thin film is formed. The final thickness and properties of the resulting coating depend on the solution viscosity, drying rate, surface energy of the solution and the substrate, the adhesion energy between the solution and the substrate, surface tension, as well as the spin speed and spinning time. For ZnO thin films, layers typically range from 50 to 600 nm. The primary advantage of the spin coating technique is the rapid, inexpensive, and precise coating of the substrate. Furthermore, it can be applied to a wide range of organic, inorganic, and composite materials. One of the main disadvantages of spin coating is the substrate size limitation: as the surface area increases, it becomes increasingly difficult to achieve high rotational speeds, which complicates the formation of a thin film [64,75,76,77,78]. A schematic of the technique is presented in Figure 5.

The spin coating technique is successfully utilized in processes for the rapid fabrication of zinc oxide coatings. In publication [79], the authors obtained a layer with a thickness of 56.3–83.0 nm using a precursor solution of anhydrous zinc acetate dissolved in a mixture of ethyl alcohol and monoethanolamine. Publication [80] demonstrated that thin-film gas sensors can be produced using the spin coating technique. The resulting films exhibited excellent adhesion and stability. Additionally, they enabled the detection of low NO_2_ concentrations (down to 5 ppm) with a sensitivity of 4.1. Spin coating also enables the fabrication of thin-film coatings with high transparency. In publication [81], the authors obtained a thin film with a transmittance of up to 80% using zinc acetate, ethanol, and stearic acid. Moreover, depending on the annealing temperature, the optical bandgap ranged from 3.69 to 3.82 eV.

Magnetron sputtering is a prominent technique for depositing zinc oxide thin films. This technique belongs more broadly to the class of physical vapor deposition (PVD) methods and is a plasma-assisted process. The process takes place in a vacuum chamber using an inert gas (typically argon), which is ionised by means of plasma. Positively charged argon ions are attracted to a negatively biased target, which acts as the cathode, and bombard its surface, causing the release of material particles. In magnetron sputtering, a magnet assembly is positioned near the target to create a magnetic field that confines the movement of electrons and charged particles. This increases the plasma density and consequently, the sputtering yield. The particles released from the target travel toward the substrate and rapidly deposit onto it, forming a thin coating. Often, a negative bias is applied to the substrate to attract positive ions and improve thin-film densification and adhesion. It should first be noted that magnetron sputtering offers numerous advantages, making it a highly desirable thin-film deposition technique for a wide range of applications [82,83,84,85].

**Figure 6:**
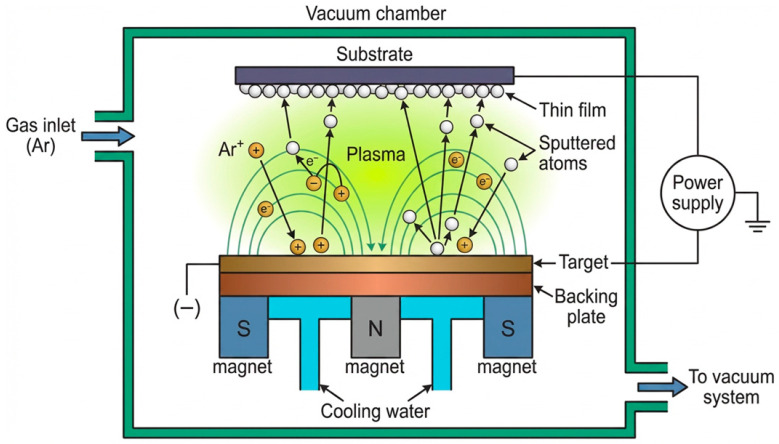
Schematic representation of the principle of magnetron sputtering of thin films (based on [84,87,88]).

The technique is characterized by high deposition rates with a minimal increase in substrate temperature, ensuring excellent coating adhesion to the substrate, and allowing for the coating of complex components. Furthermore, it enables the deposition of a wide variety of materials (metals, alloys, and oxides), allowing the creation of single-layer, multilayer, and composite coatings. The resulting films also exhibit high purity and uniformity; most importantly, from an industrial production perspective, the process is highly reproducible. For ZnO thin films, layers typically range from 100 to 500 nm. However, the technique also has drawbacks, including plasma instability, the requirement for thin targets (which increases the frequency of their replacement), limited capability for rapid sputtering of strongly magnetic materials at low temperatures, and low target utilization efficiency due to non-uniform erosion [64,82,83,84,85,86,87,88]. A schematic of the method is presented in Figure 6.

Magnetron sputtering enables the fabrication of high-transparency ZnO thin films. In publication [89], the authors obtained, depending on the parameters used, a zinc oxide coating with transparency ranging from 72 to 82%. The optical bandgap, however, varied between 3.18 and 3.23 eV. It was demonstrated that as the deposition power increases (from 500 to 800 W), the bandgap increases while the transparency decreases. Favorable optoelectrical properties were also demonstrated in publication [90]. The authors obtained zinc oxide thin films characterized not only by excellent transparency (>90%) in the visible light range but also by low resistivity (≈10^−2^ Ω·cm). Furthermore, the authors of publication [91] demonstrated that within the temperature range they investigated (100–300 °C), temperature has no effect on the transparency of the resulting coatings. For the obtained films, they observed a high transparency of 80–90% in the visible light range. In contrast, a slight decrease in the bandgap (from 3.64 to 3.56 eV) was shown as the process temperature increased.

The magnetron sputtering process can be successfully utilized to produce porous zinc oxide thin films intended for the photocatalytic degradation of organic pollutants [92]. Additionally, ZnO thin films are effectively employed in the production of memristors due to their stable resistance, uniformity, relatively low processing cost, low impurity content, and low bias requirements for SET and RESET voltages [93]. It has also been demonstrated that piezoelectric sensors can be obtained using magnetron sputtering. Furthermore, this technique enables the fabrication of layered piezoelectric sensors. In study [94], the authors successfully developed a sensor featuring a ZnO layer that effectively monitored pressure changes.

Among other vacuum deposition techniques, chemical vapor deposition (CVD) stands out. The fundamental difference between CVD and PVD methods is that CVD utilizes chemical phenomena during deposition, whereas PVD relies on physical phenomena. The difference also arises from the temperature ranges employed during deposition: for PVD, the range is from room temperature to 500 °C, while for CVD it is 900–1400 °C [95,96,97]. Prior to the CVD process, the interior of the vacuum chamber and the substrate (cold substrates are used in specific cases) are heated to a specified temperature. Subsequently, reactive (precursor) gases are pumped into the chamber. As the gases flow through the reactor, a homogeneous reaction occurs, yielding intermediate products and gaseous by-products [98,99]. Consequently, both the precursor gases and the reaction intermediates adsorb onto the substrate and diffuse across its surface. This leads to a heterogeneous reaction at the gas–solid interface, resulting in the formation of a thin film of material through nucleation, growth, and coalescence. Simultaneously, by-products are formed during both the homogeneous and heterogeneous reactions. The final step involves removing reaction products and unreacted substrates remaining in the chamber that are not bound to the substrate. Gas-phase reactions occur when the temperature is sufficiently high or when additional energy is introduced, for example, via plasma. Materials with varying properties can be fabricated by altering CVD process parameters, such as the substrate type, substrate temperature, chemical composition of the reactant gas mixtures, and total gas pressure [99,100].

**Figure 7:**
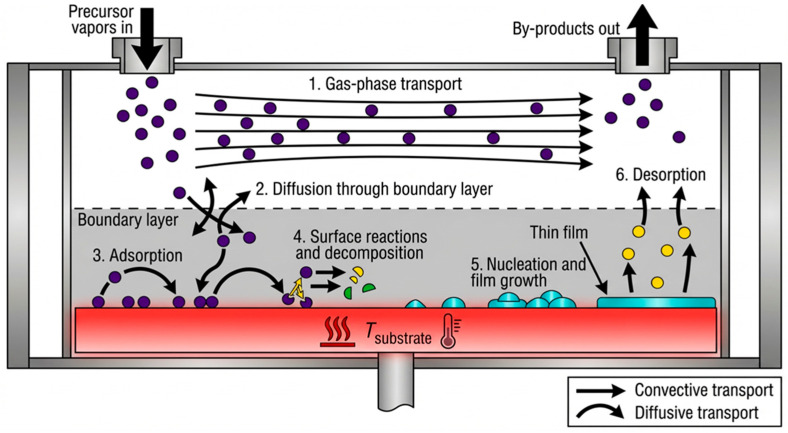
Schematic representation of thin film formation during the CVD process (based on [97,98,103]).

Similar to the previously discussed MS method, the resulting coatings exhibit excellent purity. Other advantages of the process include high efficiency, high-quality coatings, a wide choice of precursors and substrates, a rapid (and easily adjustable) growth rate, and minimal reaction by-products, which can be removed after the process is completed. However, the process also has drawbacks, including the generation of chemical waste, thermal stresses, the toxicity, explosivity, or corrosivity of precursor gases, and the need to purchase expensive, complex equipment. This process produces thin ZnO layers, typically 200–800 nm thick [64,95,96,97,98,99,100,101,102,103]. A schematic of the CVD method is presented in Figure 7.

In the case of zinc oxide coating deposition via CVD, two of its variants are frequently utilized: low-pressure chemical vapor deposition (LPCVD) and plasma-enhanced chemical vapor deposition (PECVD). LPCVD is performed at pressures ranging from 0.1 to 10 Torr and temperatures from 200 to 800 °C. In contrast, PECVD utilizes plasma to provide the energy required to drive the chemical reaction. The process pressure ranges from 2 to 10 Torr, while the temperature is between 200 and 400 °C [104]. There is also increasing discussion regarding the use of aerosol-assisted chemical vapor deposition (AACVD). The operating principle of AACVD is based on atomising a metal precursor dissolved in an organic solvent into fine aerosol droplets, which are then transported by a carrier gas, typically nitrogen, into a heated reaction chamber. There, the precursor-containing aerosol droplets evaporate, followed by homogeneous or heterogeneous chemical reactions that yield the desired material as a powder or a layer on the substrate surface. AACVD can operate at temperatures from 100 to 550 °C, and coatings are most commonly deposited under atmospheric pressure conditions [105,106].

The authors of publication [107] demonstrated that by using the CVD method, zinc oxide can be deposited on a glass substrate to function as a waveguide sensor. The authors obtained a zinc oxide coating by reacting diethylzinc with oxygen and water. The results show that the ZnO waveguide sensor provides good sensitivity in detecting various concentrations of glucose solutions. The maximum sensitivity was determined to be 1.09 dB. The obtained results indicate a potential application for chemical leak detection. Work [108] highlights the possibility of obtaining zinc oxide thin films on a polymer substrate; in this case, the coating was deposited on a polyethylene terephthalate (PET) surface. In this process, diethylzinc (C_2_H_5_)_2_Zn (DEZ) serves as the metal–organic precursor, undergoing plasma-induced oxidation within an Ar/O_2_ atmosphere to form stoichiometric zinc oxide. Using the LPCVD technique and varying the power supply in the range of 200 to 300 W, samples were obtained featuring the lowest electrical resistivity of 1.8 × 10^−4^ Ω·cm. The efficiency was determined to be 5.68%, while the transparency for all samples obtained exceeded 80%. The authors point out that the achieved parameters enable the application of a zinc oxide coating on a PET substrate in dye-sensitized solar cells (DSSCs).

The authors of publication [109] fabricated zinc oxide coatings on a silicon and glass substrate using the PECVD technique. The precursor used in this study was zinc acetylacetonate Zn(acac)_2_ with the chemical formula (Zn(C_5_H_7_O_2_)_2_·xH_2_O)_2_. The powder sublimation process was carried out at 150 °C using a heating system with precise temperature control, after which the resulting vapors were transported to the plasma chamber in an argon flow (4 sccm). At the same time, reaction oxygen was fed into the system at a flow rate of 20 sccm. Three samples were prepared at substrate temperatures of 200, 300, and 400 °C, respectively. It was demonstrated that as the substrate temperature increased, the transparency of the resulting thin films increased, reaching 70–80%. The same trend was observed for the obtained bandgap values, which ranged from 3.29 to 3.35 eV. In summary, the resulting films exhibit good transparency and semiconducting properties.

Study [110] presents research on the use of the AACVD technique to fabricate zinc oxide thin films intended as sensor elements for measuring ethanol and nitrogen dioxide concentrations. Thin films of ZnO were deposited on alumina ceramic and glass substrates. The precursor solutions for particulate (ZnO-c) and pyramidal (ZnO-p) ZnO films were prepared by dissolving 0.025 g of ZAD in 10 mL of methanol and a mixture of 0.1 g of ZAD and 0.33 mL of acetic acid in 10 mL of methanol, respectively. It was demonstrated that the resulting coatings exhibited very high sensitivity: 41 to 1 ppm NO_2_ and ~236 to 100 ppm ethanol. In contrast, the authors of publication [111] demonstrated the suitability of this method for fabricating zinc oxide thin films for photovoltaic applications. The mononuclear zinc single-source precursor was obtained by reacting Zn(OAc)_2_·2H_2_O with N,N-dimethylaminoethanol (dmaeH) in a toluene medium. This mixture was stirred intensely at ambient temperature overnight, followed by cannula filtration. The resulting transparent solution was then aged at −10 °C for 5 days. This complex served as the starting material for the growth of ZnO thin films on conductive FTO substrates. Prior to the procedure, the substrates were ultrasonically cleaned in multiple solvents and pre-equilibrated in the tube furnace for 10 min. The deposition involved aerosolizing a solution of 50 mg of the precursor in 10 mL of anhydrous toluene using an ultrasonic humidifier, with compressed air (150 cm^3^ min^−1^) acting as the carrier gas to transport the mist into the 400 °C reaction zone. The obtained films featured a bandgap of 3.45 eV and a broad absorption spectrum covering the range from 320 to 570 nm.

The last of the widely used vacuum techniques in the thin-film deposition process is atomic layer deposition (ALD). This technique is a variant of the CVD method, in which a chemical process is used to form thin films (as well as isolated nanoparticles) based on self-limiting reactions at the gas–solid interface. The self-limiting growth mechanism allows for precise control of the deposited layer thickness, which is particularly useful when high-precision coatings are required. Deposition occurs cyclically, with the thickness of the material deposited in each cycle ranging from less than 0.2 to 12 nm [112,113,114].

Most ALD reactions utilize a precursor and a reagent. The precursor is introduced into the chamber to form a monolayer on the substrate surface. Then, the reagent application is preceded by purging the chamber with an inert gas to remove chemical reaction by-products and residual gas that was not deposited on the sample surface. In the next stage, the reagent is pulsed into the chamber to react with the precursor. In this process, the metal-containing precursor reacts with surface groups on the substrate and, in a chemisorption state, interacts with a co-reactant. As a result of the occurring reactions, the desired layer is deposited onto the substrate surface. ALD is used for depositing thin films (<100 nm). The ALD technique can be applied across a wide temperature range, from room temperature to even 700 °C. However, the process is typically conducted at temperatures below 350 °C. The ALD technique enables the production of uniform, conformal, and atomically smooth layers with a well-controlled chemical composition. Layers can be deposited on a wide spectrum of substrates (metals, glass, polymers, oxides, etc.) [114,115,116,117,118,119,120,121,122].

**Figure 8:**
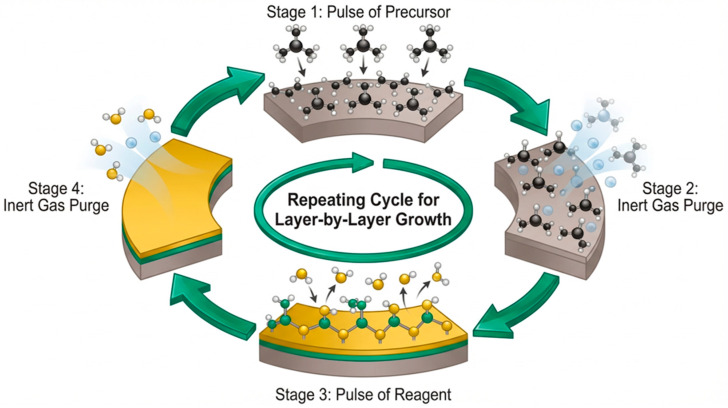
ALD process diagram films (based on [113,122]).

The technique is effective for coating objects with complex shapes and enables the fabrication of multilayer coatings. Furthermore, it is generally characterized by simple design, process scalability, and the potential for industrial implementation in mass production. However, this technique requires expensive equipment, is time-consuming, and the reaction mechanisms may not be fully understood based on experiments alone. Additionally, there is a limited number of available gaseous precursors, while the complexity of reaction mechanisms and the difficulty of validating experimental models affect the predictability of simulations and pose a challenge in scalability processes [112,113,114,115,116,117,118,119,120,121,122]. A schematic of the ALD process is presented in Figure 8.

Atomic layer deposition is a unique fabrication technique that yields ZnO layers with a relatively high free-electron concentration, typically well above 10^19^ cm^−3^. In combination with a low effective electron mass of approximately 0.24 m_e0_, this results in a high carrier mobility [123]. In publication [124], the authors investigated the effect of substrate temperature (from 55 to 135 °C) on the properties of the resulting ZnO layers. In study [124], the authors investigated the effect of substrate temperature (ranging from 55 to 135 °C) on the properties of ZnO layers deposited on borosilicate glass, silicon wafers, and PET. DEZ and deionized water vapor (H_2_O) were used as precursors due to their high vapor pressures, strong reactivity, and wide deposition temperature range. Nitrogen was used as a carrier gas for the precursor and reagent, and as a diluent in the reactor. The results indicate that for each investigated temperature range, the transparency of the obtained layers exceeds 80%, and the resistivity is within the range of 3.2–9.0 × 10^−3^ Ω·cm. The authors demonstrated that transmittance, bandgap, refractive index, and extinction coefficient change little, whereas resistivity decreases slightly with increasing temperature. Furthermore, grain growth occurs as the temperature increases. Favorable optoelectrical properties were also demonstrated by the authors of work [125]. The layers were deposited on SiO_2_ and glass substrates at temperatures ranging from 46 to 141 °C using DEZ, H_2_O, and nitrogen at a flow rate of 500 sccm. The resulting layers featured transparency, reaching approximately 90%; the bandgap was in the range of 3.23–3.28 eV; and the carrier concentration was approximately 10^19^ cm^−3^ (at 141 °C). It was also shown that increasing the temperature accelerated the coating growth rate from 0.16 to 0.28 nm/cycle. The properties of the resulting ZnO thin films are also significantly influenced by the substrate on which they were deposited. This was demonstrated by the authors of publication [126], in which the layer deposition process was conducted on silicon and aluminum(III) oxide substrates at variable temperatures (100–300 °C). A thin ZnO coating was formed as a result of a double-exchange chemical reaction between deionized water and DEZ. The layer deposited on silicon was characterized by a lower stress value and dislocation density compared to the layer on aluminum(III) oxide at the same temperature. Conversely, the layer deposited on aluminum(III) oxide exhibited a higher electron concentration than the layers deposited on silicon. On the other hand, electron mobility was higher on the silicon substrate. These results indicate the importance of substrate selection for the future applications of the ZnO layer.

The ALD technique is successfully utilized to produce zinc oxide thin films intended as components for sensors measuring NO_2_ concentration. The authors of publication [127] obtained a coating with a thickness of approximately 18 nm, which exhibited an exceptionally high sensitivity of 2100 to 10 ppm NO_2_ at 300 °C. This value is among the highest reported to date for zinc oxide-based gas sensors. Under ultraviolet radiation, the sensitivity increases to 10.000 and the recovery time is shortened from 400 to just 2 s. These coatings were deposited on Si/SiO_2_ substrates equipped with prepatterned interdigitated Pt electrodes, using diethylzinc, deionized water, and nitrogen as precursors. Beyond gas sensing, thin ZnO layers also play a critical role in the fabrication of thin-film transistors. The authors of publication [128], using the ALD technique, obtained a layer with a thickness of ≈15 nm characterized by very high field-effect and intrinsic mobility (µFE/µo) values of 85/140 cm^2^·V^−1^·s^−1^, respectively. Importantly, the ZnO thin film acting as a transistor was integrated with RRAM memory based on a 1 kbit (32 × 32) 1T1R-type matrix, demonstrating functionality in RRAM switching. The fabrication process consisted of three main stages. First, a Ti/Pt bottom gate electrode was deposited onto a SiO_2_ substrate using electron beam evaporation (EBE). This was followed by the ALD process of a HfO_2_ gate dielectric at 250 °C, formed as a result of the reaction of tetrakis(ethylmethylamido)hafnium (TEMAH) with H_2_O. Finally, a thin active channel layer of ZnO was deposited by ALD at 150, 200, and 250 °C via the reaction between diethylzinc (DEZ) and deionized water vapor.

Table 2 summarizes the methods discussed. The comparative analysis of diverse ZnO thin-film deposition techniques reveals a fundamental correlation between the governing physical or chemical mechanisms and the resulting structural precision, as synthesised from a technical review of current methodologies. High-vacuum methodologies, specifically ALD and MS, represent the state of the art in thickness control. ALD achieves sub-nanometer resolution through self-limiting surface reactions, making it indispensable for high-performance transistors and sensors despite its temporal and financial constraints, whereas MS offers high purity and industrial scalability, with thickness controlled by linear power and time relationships. Conversely, atmospheric pressure techniques such as CBD and SP prioritise cost-effectiveness and operational simplicity by eliminating the need for complex vacuum systems, though they often exhibit diminished reproducibility and lower film density due to their high sensitivity to minor fluctuations in pH, temperature, and precursor atomization. Spin-coating provides a reliable intermediary for laboratory-scale fabrication on flat substrates, yet it remains hindered by excessive material wastage exceeding 90%; by contrast, CVD variants deliver high-quality films at the expense of managing precursor toxicity and significant thermal stresses. Ultimately, selecting a synthesis pathway requires a strategic trade-off between the required optoelectronic performance, such as carrier mobility and transparency, and the logistical complexities of the operating environment, with future advancements directed toward low-temperature, environmentally sustainable processing to reconcile high-quality output with industrial feasibility.

Beyond structural control, the thermal requirements of these methods dictate their compatibility with various substrates, where low-temperature processes like ALD and Magnetron Sputtering enable the high-quality deposition of ZnO onto heat-sensitive flexible polymers such as PET. Furthermore, the structural integrity of ZnO thin films, especially those fabricated via solution-based routes, is typically enhanced by post-deposition annealing, which improves crystallinity and minimizes intrinsic defect density. The resulting surface morphology—ranging from the characteristic porosity of sprayed layers to the atomic-scale smoothness of ALD-grown films—directly determines the intrinsic optical transparency and charge-carrier dynamics, thereby establishing the baseline performance for all subsequent functional modifications.

## 3. Doped ZnO Films

To enhance the properties of zinc oxide, doping with other elements is frequently employed, utilizing a broad spectrum of methods. The following chapter presents the methods described in Chapter 2, which are successfully utilized in the processes of doping ZnO thin films with specific elements.

The authors of publication [129] demonstrated the positive impact of tin doping on ZnO thin films using the spray pyrolysis method. It was shown that ZnO:Sn films exhibit a significant reduction in crystallite size and increased absorption, while the bandgap varies between 3.28 and 3.21 eV. A positive doping effect was also demonstrated by the authors of [130], who fabricated UV sensors based on ZnO:Sn thin films with a tin content ranging from 1 to 10 at.%. As the authors indicate, despite the highly variable tin content in the surface layer, no significant changes were observed in the fundamental optoelectrical properties. However, the resulting films were characterized by high sensitivity and photoresponse to both monochromatic and solar UV radiation. Spray pyrolysis also enables doping with multiple elements. The authors of publication [131] doped ZnO thin films with tin (1 at.%) and cobalt (0–1 at.%). They demonstrated an increase in the bandgap from a value of 3.25 eV for ZnO to ≈3.30 eV for ZnO:Sn:Co (1%:0.5%). The ZnO:Sn:Co samples doped in the aforementioned proportion exhibited the lowest resistance value of 1.96 × 10^−2^ Ω·cm, as well as the highest figure of merit (FOM) of 1.41 × 10^−4^ Ω^−1^. The obtained parameters of the doped ZnO layer indicate its promising use as a TCO for optoelectronic applications.

The spray pyrolysis process also enables the doping of ZnO thin films with elements such as: Cu [132], Al [133], Ce [134], Ag [135], Mg [136], Ni [137], Zr [138], Fe [139], Ga [140] and In [141].

The authors of publication [142] used the CBD method to obtain a nickel-doped (1–4%) ZnO layer on a glass substrate. Research results indicate that nickel doping shifted the absorption edge, and the bandgap varied from 3.17 to 3.25 eV. Notably, the bandgap decreased as the Ni concentration increased. Magnetic property studies showed that the highest values for remanent magnetization (Mₘ) and coercive field (Hc) were obtained for the ZnO:Ni layer containing 2% Ni. The values of Mₘ and Hc reached 0.38 emu/cm^3^ and 180 Oe, respectively. The authors point to the potential application of the resulting layer in spintronics, where both magnetic and optical properties are essential. This method can also be utilised to dope a ZnO layer produced by a different method. In study [143], the authors used the CBD method to dope a ZnO thin film obtained via spray pyrolysis with nickel (0–8%). Investigations of the doped layers revealed that the best optoelectrical properties were achieved for ZnO:Ni containing 2% Ni, consistent with the previous study. The sample featured an average bandgap value of 3.12 eV, a low resistivity of 6 × 10^−4^ Ω·cm (undoped sample 3.5 × 10^−3^ Ω·cm), and an FOM of 22.71 × 10^−6^ Ω^−1^. It was also shown that the highest transparency was achieved for ZnO:Ni with 2% nickel, reaching ~80%, and that this value decreased with further increases in nickel content.

In addition to nickel, the CBD method enables the doping of ZnO thin films with elements such as: Fe [144], Mn [145], Cd [146], Al [147], Mg [148], Na [149], Cu [150], K [151], Sn [152], and Ag [153].

To improve the performance of ZnO thin films, the spin coating technique is also utilized. The authors of publication [154] investigated the effect of magnesium doping on zinc oxide thin films used in solar cells, using the spin coating technique. As indicated by the researchers, the ZnO:Mg layer exhibited an energy conversion efficiency similar to that of the undoped layer. The precursor was zinc acetate dihydrate, and the solvent was absolute ethanol (C_2_H_5_OH). However, Mg doping increased device stability, reduced recombination losses, and improved charge transport. Similar results were obtained by the authors of publication [155]. They produced pure ZnO and ZnO:Mg layers at various concentrations (0–3 wt.%) and annealing temperatures (300–600 °C). Deposition was performed on silicon and quartz substrates. They demonstrated that grain size increases with increasing temperature. The sample containing 2 wt.% magnesium featured the smallest grain size. Optical characterization showed that the transmittance of the ZnO:Mg thin films increased from 74.5% (undoped ZnO) to approximately 89% (highest value for 2% Mg), and the optical bandgap energy increased from 3.24 to 3.56 eV. The doped layers exhibited a higher refractive index compared to the undoped ZnO layers. The spin coating technique also enables doping with multiple elements in a single process. The authors of publication [156] doped ZnO thin films with magnesium and copper. The ZnO and ZnO:Mg:Cu thin films were deposited on a glass substrate. The obtained results indicate high transparency for the resulting layers, both doped and undoped, on the order of 85–90%. It should be noted that the results for ZnO lie at the lower end of the specified range, while those for ZnO:Mg:Cu are closer to the upper range. The bandgap values for ZnO and ZnO:Mg:Cu were 3.34 and 3.59 eV, respectively. Furthermore, it was shown that Mg and Cu doping led to a reduction in crystallite size, surface smoothing, and enhanced photoluminescence.

In addition to the previously discussed elements, namely Mg and Cu, the spin coating technique also enables the doping of ZnO thin films with elements such as: Al [157], Ga [158], In [159], Ag [160], Mn [161], Co [162], Li [163], Sn [164], Ni [165], and Nb [166].

The authors of publication [167] demonstrated the positive impact of tantalum doping on ZnO thin films produced by magnetron sputtering. During the process, various sputtering powers for the Ta_2_O_5_ target were applied, namely 30, 35, 40, and 45 W. The researchers showed that as the sputtering power increased, the tantalum concentration in the surface layer and the bandgap value also increased (from 3.27 to 3.51 eV). The authors also pointed out a very important characteristic of tantalum: its solubility limit in ZnO is approximately 5–6 at.%, and above this value, the number of grain boundaries and defects increases, which translates into a decrease in carrier mobility and concentration. The positive effect of tantalum doping on ZnO thin films was also shown in a study [168]. The authors deposited ZnO and ZnO:Ta (0.1 at.%) thin films on a glass substrate. By modifying the deposition time (from 10 to 25 min), the researchers obtained doped thin films with thicknesses of 430, 670, 880 and 1095 nm, respectively. The carrier concentration for ZnO was 1.1 × 10^19^ cm^−3^, while the ZnO:Ta layers showed an increase in carrier concentration with increasing layer thickness, reaching the highest value for the 1095 nm thick sample—4.7 × 10^19^ cm^−3^. All obtained samples showed very good transparency at approximately 80%, while the ZnO layer exhibited the highest average reflectance of about 50%, and the lowest value of 35% was observed for the 1095 nm thick doped layer. The bandgap for ZnO was 3.19 eV, while for the ZnO:Ta layers, the following results were obtained with increasing layer thickness: 3.17, 3.30, 3.31 and 3.22 eV. MS also enables the doping of ZnO coatings with several elements in a single process. An example of this is study [169], which used magnesium (3 at.%) and fluorine (6 at.%) as dopants. The coatings were deposited on a glass substrate and then annealed at 400 °C. The resulting ZnO:Mg:F layer featured a resistivity of 1.27 × 10^−3^ Ω·cm, while the highest FOM reached 1.18 × 10^−2^ Ω^−1^.

In addition to the previously discussed elements Ta, Mg, and F, it has been demonstrated that the MS technique can be successfully used to dope ZnO thin films with elements such as: Al [170], Ga [171], Nd [172], In [173], Ag [174], Sn [175], Mn [176], Cu [177], N [178] and Gd [179].

The authors of publication [180] demonstrated the positive impact of aluminium doping on ZnO thin films prepared by AACVD using nitrogen as the carrier gas. The substrate was soda–lime glass; the process temperature was 400 °C; and the post-deposition annealing was carried out at 450 °C. The authors systematically varied the Al doping concentration from 0 at.% to 20 at.% to investigate its effect on coating properties. Key findings include the fact that increasing the Al concentration led to decreases in crystallite size and dislocation density, and a slight increase in layer thickness. An increase in the bandgap from 3.21 eV for ZnO to 3.33 eV for ZnO:Al (20 at.%) was also shown, exhibiting the Burstein–Moss effect and increased UV absorbance at lower doping levels. The positive impact of aluminium doping on ZnO using the AACVD technique was also demonstrated by the authors of publication [181]. In this work, oxygen was used as the carrier gas, unlike the previously discussed study. The researchers varied the aluminium concentration from 5 to 20 at.%, and, as in the previously discussed work, the process temperature was 400 °C, and the annealing temperature was 450 °C. The key result of the research conducted was an increase in the bandgap from 3.57 eV (ZnO) to 3.59 eV at a 5% Al concentration. At higher aluminium content, a decrease to 3.4 eV at 20% occurs. Furthermore, it was shown that excessive doping above the critical threshold leads to a decrease in carrier mobility and an increase in resistivity due to increased lattice disorder and enhanced scattering. The AACVD technique can also be successfully applied to dope the ZnO layer with multiple elements. The authors of work [182] synthesised ZnO:F, ZnO:Mo, and ZnO:Mo:F thin films. Their research focused on optimising dopant concentrations and deposition temperatures. The deposition temperature was between 450 and 550 °C, while approximately 4.6 at.% Mo and 1.5 at.% F were identified as the optimal concentrations for single doping, and 5.7 at.% Mo and 1.3 at.% F were identified as the optimal concentrations for co-doping. They observed that the doping process significantly improved the optoelectrical properties, reducing the resistivity from ~10^2^ Ω·cm for ZnO to 5.084 × 10^−3^ Ω·cm for ZnO:Mo:F, while maintaining high visible light transmittance of 75–85%.

In addition to the previously discussed elements Al, Mo, and F, it has been demonstrated that the CVD method can be successfully used to dope ZnO thin films with elements such as: B [183], Li [184], Ga [185], Sb [186], Mg [187], Cd [188], Mn [189], Ni [190] and Cu [191].

The authors of publication [192] demonstrated the positive effect of aluminium doping on zinc oxide thin films prepared by ALD. The authors controlled the aluminium concentration between 0.3 and 6.7 at.%, while maintaining a constant layer thickness of approximately 180 nm. Consequently, by applying 1.1 at.% Al, a high-quality thin film was obtained, characterized by a low electrical resistivity of 6.3 × 10^−4^ Ω·cm. This layer also exhibits good optical transmittance above 85% in the visible range, a high FOM of 6.4 × 10^−3^ Ω^−1^, and a high degree of c-axis orientation. The positive influence of Al doping on the optoelectrical properties of the ZnO layer was also shown by the authors of work [193]. In this study, ZnO:Al thin films deposited by ALD at 180 °C were investigated. After the process was completed, the samples were exposed to UV radiation and ozone for 30 min at room temperature or subjected to thermal annealing at 600 °C for 30 min in an air atmosphere. Exposure of the thin films to UV radiation and ozone reduced the resistance from 66 Ω·cm (as deposited) to 56.12 Ω·cm. A similar effect was observed with thermal annealing, where the resistance reached 59.52 Ω·cm. The ALD method enables simultaneous doping with several elements in a single process. In publication [194], the authors doped the ZnO thin film with aluminium and fluorine. The process was carried out at 200 °C. In the case of the ZnO:Al layer, the Al content varied from 1.67 to 10%, whereas for ZnO:Al:F, the Al concentration was kept constant at 5%, while the fluorine concentration varied from 0.1 to 10%. The ZnO:Al layer exhibited its optimal optoelectronic properties at a 5% aluminium concentration. The layer reached a low resistivity of 1.36 × 10^−3^ Ω·cm and high transparency (>80%). For the ZnO:Al:F layer, a slight increase in resistivity was obtained compared to the discussed ZnO:Al layer; nevertheless, the film is characterized by good optical properties, high transparency, and a bandgap value between 3.53 and 3.58 eV (ranging from 3.32 to 3.75 eV for ZnO:Al).

In addition to the previously discussed elements, it has been demonstrated that the ALD technique can be successfully used to dope ZnO thin films with elements such as: Co [195], Ge [196], Cu [197], Hf [198], Fe [199], Ni [200], Sn [201], In [202], Ga [203], and Zr [204].

Table 3 summarizes the multifaceted correlation between specific deposition methodologies and strategic doping architectures, providing a comprehensive benchmark for current advancements in ZnO thin-film engineering. The data reveal that the integration of diverse dopants spanning d-block transition metals (Ni, Co, Mo, Ta), post-transition elements (Sn, Al), and alkaline earth metals (Mg) is intrinsically linked to the governing physical and chemical mechanisms of the synthesis pathway and the resulting defect chemistry of the ZnO lattice. High-vacuum techniques, such as ALD and MS, continue to define the upper limits of structural and electronic precision by facilitating epitaxial-like growth and minimising intrinsic scattering centres. Specifically, Al-doped ZnO via ALD achieves a record FOM of 6.4 × 10^−3^ Ω^−1^ and superior c-axis orientation due to the atomic-scale control of self-limiting surface reactions, while Ta-doping through MS enables exceptional carrier density control (4.7 × 10^19^ cm^−3^), which is essential for high-performance TCO applications requiring maximized dopant activation without compromising the crystalline integrity of the film.

In contrast, atmospheric and solution-based routes prioritize functional versatility and cost-effectiveness without necessarily sacrificing optoelectronic quality. SP and AACVD leverage synergistic co-doping (Sn + Co and Mo + F, respectively) to reconcile high transparency with reduced resistivity, effectively bypassing the thermodynamic solubility limits of single-element dopants to offer viable vacuum-free alternatives for industrial-scale optoelectronics. Furthermore, the table highlights a significant expansion in the functional scope of ZnO. By contrast, Spin Coating with Mg-doping focus on bandgap engineering (Eg up to 3.56 eV) to effectively broaden the solar window and improve charge carrier dynamics for solar cell stabilization; CBD with Ni-incorporation facilitates the seamless integration of magnetic and optical properties, achieving a high coercivity of 180 Oe, indicative of a successful diluted magnetic semiconductor (DMS) phase for spintronic transport.

Ultimately, this comparative synthesis demonstrates that the optimization of ZnO thin films is no longer dictated solely by the deposition technique, but by the precise synergy between the growth environment and the dopant’s electronic configuration, as well as by the suppression of non-radiative recombination pathways. While vacuum-based methods remain indispensable for subnanometer resolution and maximum carrier mobility, the emergence of high-performance co-doped systems via atmospheric processes marks a strategic shift toward sustainable, large-area electronic fabrication and “green” chemical processing. Future progress necessitates balancing these record-breaking optoelectronic parameters with the logistical demands of device integration, long-term and complex mitigation of ionized impurity scattering in heavily doped systems.

## 4. Discussion Summary and Outlook

Zinc oxide has established its position as a versatile material, as evidenced by the dynamic growth of its market. This material has found applications in fields such as nanostructures, chemical sensors and biosensors, photocatalysis, medicine, vulcanization, the cosmetics industry, agriculture, the food industry, and photovoltaics. In the semiconductor field, ZnO demonstrates particular potential in optoelectronic applications, notably as a transparent conducting oxide, in solar cells, memristors, gas sensors, thin-film transistors, and as a photocatalyst. The development of ZnO thin-film fabrication methods has enabled precise control over their optical and electrical properties, tailored to specific applications. Table 4 summarizes selected properties of the thin ZnO film obtained using the deposition techniques discussed in this article. Based on the data presented, it is clear that different properties can be obtained using a given deposition technique. This means that a particular method can be used to realize the function intended for the thin ZnO layer. The primary novelty of this comprehensive review lies in the systematic juxtaposition of these divergent synthesis pathways, revealing a “property-by-design” paradigm that is critical for next-generation optoelectronics. Specifically, the data demonstrates that vacuum-based and gas-phase routes, such as Chemical Vapor Deposition and Atomic Layer Deposition, represent the pinnacle of electrical performance, achieving carrier mobilities up to 100 cm^2^/V·s and ultra-low resistivity values reaching 10^−6^ Ω·cm. This dramatic enhancement—up to four orders of magnitude superior to solution-processed films—is attributed to the high-purity, defect-free crystalline growth facilitated by controlled environments, which minimize grain boundary scattering.

Optically, the study highlights a versatile tunability of the band gap, ranging from 2.59 eV in Chemical Bath Deposition to 3.7 eV in Spray Pyrolysis and CVD. This broad spectral flexibility, coupled with the exceptional visible-light transparency of ALD and SP (up to 95%), underscores the potential of ZnO as a universal transparent conducting oxide. Furthermore, the structural precision offered by ALD, which allows for ultra-thin layers (as low as 5 nm), provides a unique solution for the miniaturization of nanotransistors and quantum devices, a feat that remains challenging for more robust, high-throughput techniques like Magnetron Sputtering. By bridging the gap between fundamental synthesis parameters and macro-scale functional outputs, this work serves as a strategic roadmap for researchers. It clarifies the trade-offs between processing cost and material quality, ultimately providing the necessary framework for selecting deposition methods to optimise applications in solar cells, memristors, and advanced biosensors.

The future of research on ZnO thin films lies in a comprehensive understanding of deposition mechanisms, which will facilitate enhanced reproducibility and control over film properties. It is crucial to develop fewer toxic precursors and advance material recycling methods for processes such as CBD and CVD to mitigate environmental impact. Doping ZnO with various elements further enhances its optoelectronic, magnetic, and structural properties, paving the way for applications in diverse modern technologies. Optimizing synthesis and modification parameters should yield materials with superior optical, electrical, and mechanical characteristics. A particularly promising direction is the development of applications in advanced optoelectronic and photovoltaic devices, including next-generation solar cells.

A significant area of future scientific exploration will be the development of flexible and wearable electronics, necessitating the intensification of research into low-temperature techniques, such as Spatial Atomic Layer Deposition (SALD) and room temperature magnetron sputtering. These techniques enable the deposition of high-quality films on polymeric substrates without the risk of thermal degradation, while maintaining high conductivity and transparency. Furthermore, one of the most critical challenges remains overcoming doping asymmetry to achieve stable and durable p-type conductivity. However, the realization of such advanced devices is currently hindered by fundamental electronic and structural limitations. The most critical challenge remains the doping dilemma: achieving stable, durable p-type conductivity despite strong self-compensation mechanisms. Native donor-like defects, such as oxygen vacancies and zinc interstitials, spontaneously counteract intended acceptor doping, while most p-type dopants exhibit low solubility and high ionization energies, leading to deep acceptor levels and poor temporal stability. Beyond electronic asymmetry, the inherent amphoteric nature of ZnO poses significant operational hurdles; exposure to atmospheric humidity can lead to the formation of surface hydroxides and carbonates, which drastically increase contact resistance and degrade surface potential. Additionally, in the field of flexible electronics, a persistent mechanical–thermal trade-off persists; low-temperature deposition often yields films with lower crystallinity and higher densities of sub-gap states than high-temperature processes, increasing the material’s susceptibility to Negative Bias Illumination Stress (NBIS).

Furthermore, a significant physical limitation arises from the fundamental trade-off between electrical conductivity and optical transparency, primarily governed by the Burstein–Moss effect. As the carrier concentration is increased through heavy doping to reach metallic-like resistivity, the absorption edge shifts, often leading to increased parasitic absorption in the near-infrared region and enhanced ionized impurity scattering, which ultimately caps the maximum achievable charge-carrier mobility. Beyond bulk properties, a critical hurdle for device integration is precisely tuning the work function of ZnO thin films. In multilayer films, such as perovskite or organic solar cells, even slight energy-level misalignments at the ZnO interface can create significant charge-extraction barriers, drastically reducing overall power conversion efficiency. Current reliance on complex surface dipoles or interfacial modifiers to alleviate these barriers complicates the manufacturing process and raises concerns regarding the long-term chemical stability of the resulting heterojunctions. Addressing these challenges through precise defect engineering and advanced encapsulation strategies is essential for transitioning ZnO to a robust industrial standard.

This objective will allow for the construction of efficient ZnO homojunctions, high-performance light-emitting diodes (LEDs), and advanced complementary metal-oxide-semiconductor (CMOS) circuits based entirely on ZnO. In the era of sustainable development, a priority will also be the optimization of indium-free layers, such as Al-doped ZnO and Ga-doped ZnO, with a focus on their environmental stability and long-term corrosion resistance during operational life. Future research will increasingly rely on artificial intelligence (AI) and machine learning (ML) tools. The use of advanced algorithms to predict physicochemical properties from multidimensional process parameters will drastically reduce the time required to optimize them.

In conclusion, ZnO is a material of immense potential, the further development of which depends on systematizing knowledge of thin-film fabrication and modification methods, addressing current technological challenges, and expanding its range of applications in innovative technologies of the future.

## Figures and Tables

**Table 2:** Characteristics, advantages, and limitations of various ZnO deposition techniques.

Deposition Method	Process Temperature (°C)	Operating Environment	Thickness Control	Main Advantages	Main Disadvantages
Spray Pyrolysis	200–500	Atmospheric	Influenced by droplet size, spray rate, and nozzle-to-substrate distance	Simple, low cost, no vacuum required, high transparency (>95%)	Difficulty in achieving high density; material is often porous
Chemical Bath Deposition	<100	Atmospheric (solution)	Controlled by immersion time, bath temperature, pH, and precursor concentration	Lowest cost, simple equipment, ability to coat multiple elements simultaneously	High sensitivity to pH and concentration, toxic liquid waste, poor reproducibility
Spin Coating	25 (+annealing)	Atmospheric	Precisely determined by solution viscosity and centrifugal (RPM) speed	Speed, accuracy, ideal for lab tests and polymers	Significant material loss (>90%), limited to small flat substrates
Magnetron Sputtering	25–500	Vacuum	Highly repeatable; thickness is a direct linear function of power and time	High purity, excellent adhesion, industrial scalability, high deposition rate	Plasma instability; uneven target wear; expensive vacuum equipment
Chemical Vapor Deposition	100–1400 (variant-dependent)	Vacuum/Atmospheric	Controlled by gas flow rates and temperature stability; very consistent	High efficiency and film quality; wide choice of precursors	Toxicity and explosiveness of gases, thermal stress; complex apparatus
Atomic Layer Deposition	25–300	Vacuum	Based on self-limiting atomic cycles, allows for subnanometer precision	Superior uniformity and conformality, smoothness, high carrier mobility	Very slow growth rate (time-consuming), high equipment and operating costs

**Table 3:** A comparison of ZnO deposition methods in terms of their electrical, optical and application-relevant properties.

Deposition Method	Dopant	Innovation Area	Key Electrical & Optical Parameters	Synergistic Optoelectronic Impact	Novelty Benchmark
Spray Pyrolysis	Sn + Co	Optoelectronic TCO applications without vacuum	E_g_ ≈ 3.30 eV, *ρ* = 1.96 × 10^−2^ Ω·cm FOM = 1.41 × 10^−4^ Ω^−1^	Achieving low resistivity with increased bandgap for optoelectronic devices	Achieving promising TCO parameters using atmospheric spray pyrolysis for optoelectronic use
Chemical Bath Deposition	Ni	Spintronic applications: Integration of magnetic and optical properties	E_g_ = 3.12–3.25 eV, M_m_ = 0.38 emu/cm^3^, H_c_ = 180 Oe	Seamlessly integrating magnetic properties within a high-transparency TCO matrix	Reaching a high coercivity of 180 Oe in a solution-processed film, essential for spin-polarized transport
Spin Coating	Mg	Solar cell enhancement and device stability	Transmittance ≈ 89%, E_g_ = 3.24–3.56 eV	Drastic reduction in recombination losses while significantly improving charge transport efficiency	Increasing transparency to 89% and improving charge carrier dynamics compared to undoped ZnO
Magnetron Sputtering	Ta	Carrier density control via thickness and concentration	n_e_ = 4.7 × 10^19^ cm^−3^, E_g_ = 3.17–3.31 eV	Maximizing carrier concentration via precise thickness control (1095 nm) while maintaining 80% visibility	Optimizing Ta solubility (5–6 at.%) to achieve record carrier density without degrading crystal quality
Chemical Vapor Deposition (AACVD)	Mo + F	Synergistic co-doping for resistivity reduction	*ρ* = 5.084 × 10^−3^ Ω⋅cm. Transmittance 75–85%	Significant improvement in optoelectronic properties through optimized co-doping	Achieving high-visibility TCO performance via Mo + F synergy, offering a viable alternative to Ga-doped systems
Atomic Layer Deposition	AI	High-quality TCO films with c-axis orientation	FOM = 6.4 × 10^−3^ Ω^−1^, Transmittance >85%	Achieving resistivity close to ITO with record-high carrier mobility	Obtaining high-quality films with strong c-axis orientation and low resistivity at 1.1 at.% Al

**Table 4:** Comparison of methods for producing thin ZnO layers.

Deposition Method	Band Gap (eV)	Resistivity (Ω·cm)	Carrier Mobility (cm^2^/V·s)	Transmittance (%)	Film Thickness (nm)
Spray Pyrolysis	3.1–3.7	10^−2^–10^−4^	15–50	75–95	150–700
Chemical Bath Deposition	2.59–3.37	10^−2^–10^−3^	5–20	80–90	100–500
Spin Coating	3.0–3.5	10^−2^–10^−4^	10–30	85–92	50–600
Magnetron Sputtering	3.0–3.6	10^−2^–10^−4^	15–50	85–90	100–500
Chemical Vapor Deposition	3.3–3.7	10^−4^–10^−5^	50–100	85–93	200–800
Atomic Layer Deposition	3.2–3.4	10^−4^–10^−6^	60–100	90–95	5–100

## Data Availability

No new data were created or analyzed in this study. Data sharing is not applicable to this article.
